# Worse Clinical Outcomes in Acute Myocardial Infarction Patients with Type 2 Diabetes Mellitus: Relevance to Impaired Endothelial Progenitor Cells Mobilization

**DOI:** 10.1371/journal.pone.0050739

**Published:** 2012-11-30

**Authors:** Lin Ling, Yu Shen, Kun Wang, Chunying Jiang, Chunmei Fang, Albert Ferro, Lina Kang, Biao Xu

**Affiliations:** 1 Department of Cardiology, The Affiliated Drum Tower Hospital, School of Medicine, Nanjing University, Nanjing, China; 2 Department of Clinical Pharmacology, Cardiovascular Division, School of Medicine, King’s College London, London, United Kingdom; University of Padova, Medical School, Italy

## Abstract

**Background:**

Although the clinical outcome of acute myocardial infarction (AMI) in patients with type 2 diabetes mellitus (T2DM) is well established to be worse than for non-diabetic patients, the reasons for this remain unclear. We hypothesized that this may be related to impairment of bone marrow-derived endothelial progenitor cells (EPCs) mobilization.

**Methodology/Principal Findings:**

We observed short term bone marrow EPCs mobilization and long term clinical outcomes in 62 AMI patients with or without T2DM and investigated EPCs levels as well as bone marrow pathway changes in a rat model of diabetes after AMI. Patients with T2DM exhibited a delay (peak time diabetics vs. non-diabetics: day 7 vs. day 5) and a decrease in EPCs mobilization (diabetics vs. non-diabetics: 285±56/10^6^ mononuclear cells (MNCs) vs. 431±88/10^6^ MNCs, p<0.05) within one month after AMI. Plasma levels of VEGF and SDF-1α as well as of hsCRP were higher in T2DM patients. Over a mean of 2.26 years follow-up, T2DM patients exhibited a pronounced decrease in LVEF as well as an increase in clinical events. Glucose (HR 2.01, 95% CI 1.42–2.85, p = 0.008), first day EPC (HR 0.974, 95% CI 0.952–0.997, p = 0.02) and seven day EPCs (HR 0.966, 95% CI 0.945–0.988, p = 0.003) were independent prognostic variables for cardiovascular mortality. In a diabetic rat model of AMI, decreased circulating EPCs was accompanied by lower expression of phospho-Akt, phospho-eNOS, HIF, MMP-9 and MMP-9 activity in the bone marrow as well as impaired cardiac function, angiogenesis and increased left ventricle remodeling.

**Conclusions/Significance:**

Bone marrow EPCs mobilization is delayed and reduced in diabetes, with impaired HIF/p-Akt/p-eNOS/MMP-9 signaling. This is likely to contribute to the deterioration in cardiac function and worsened clinical outcome seen in patients with T2DM.

## Introduction

Although clinical outcomes of acute myocardial infarction (AMI) in patients with type 2 diabetes mellitus (T2DM) is well established to be worse than non-diabetic patients [1], [2], the reasons for this remain unclear. It has been shown that patients following AMI exhibited increased mobilization of endothelial progenitor cells (EPCs) from the bone marrow [3]. These EPCs, once mobilized into the peripheral circulation, home to infarcted myocardium and are involved in the maintenance of endothelial homoeostasis and new vessel formation [4]–[7]. Circulating EPCs level and function are predictive for prognosis following AMI, correlate with cumulative cardiovascular risk, cardiovascular mortality and atherosclerosis progression in patients with coronary artery disease [8]–[14].

Consistent evidences showed that the defect in the mobilization, recruitment and function of EPCs are hallmark features in diabetes and EPCs alterations served a pathogenic role in the development of diabetic complications. Fadini et al first proved that diabetic animals showed defective EPCs mobilization and compensatory angiogenesis in a hindlimb ischaemia-reperfusion model [15]. Besides, circulating EPCs levels are found to be negatively correlated with the proceeding of vascular associated complications of diabetes, including diabetic retinopathy, nephropathy, and wound healing [16]. T2DM are proved to be related with decreased level and impaired function of circulating EPCs, including decreased proliferation, migration abilitities and increased apoptosis, resulting in reduced capacity for angiogenesis and tissue recovery [17]–[19].

Bone marrow defective, both structural and functional, were found in diabetes mellitus, with impaired mobilization response of EPCs after various ischemia stimulations. Oikawa et al demonstrated that profound structural and morphological changes occurred in the bone marrow from mice with type 1 diabetes, with basement membrane thickening, capillary rarefaction and subsequent depletion of endothelial cells and progenitor cells [20]. EPCs mobilization following ischemic AMI requires the normal signaling within the bone marrow, including hypoxia induced factor (HIF), vascular endothelial growth factor (VEGF) and so on. The abnormal function of this pathway may result in defective EPC mobilization, leading to impairment of myocardial repair [21]–[24]. The obstruction of either the framework or the signaling pathways in the bone marrow in diabetes mellitus may interfere the bone marrow response to the ischemia induced endothelial progenitor cells mobilzaiton, thus leading to the insufficient release and homing of EPCs and subsequent increased vascular complications in diabetes mellitus. These considerations raise the possibility that the worsened outcome post-AMI in patients with T2DM may also relate to impaired bone marrow mobilization of EPCs.

In the present study we therefore examined the dynamics of EPCs mobilization in patients following AMI who either did or did not have T2DM, and related these observations to cardiovascular events and cardiac function on follow-up. In a rat model of diabetes, we investigated whether reduced HIF/VEGF/Akt/eNOS/MMP-9 signaling in the bone marrow may underlie impaired EPCs mobilization in response to AMI as compared to control (non-diabetic) rats.

## Methods

### Ethics Statement

Animal study were approved by Nanjing University Scientific and Animal Ethics Committee (Approval Number: 20080125) and were in compliance with the Chinese national regulations on the use of experimental animals. Clinical researches were approved by the Ethics Review Board for Clinical Studies of Nanjing Drum Tower Hospital (Approval Number: NDT0090029). All patients gave written informed consent and research was conducted according to the principles expressed in the Declaration of Helsinki.

### Study Subjects

We enrolled 62 patients of acute myocardial infarction, of which 31 were T2DM (DM), and 31 non-diabetic (NDM). The non-diabetic group was closely matched to the diabetic group in basic characteristics. Both groups did not receive immediate PCI but alternatively underwent later PCI or CABG (10 days or later). Male SPF 3–4 week Sprague-Dawley rats were used to induce experimental diabetes [25], [26]. After 4 weeks high-fat diet, animals were injected with streptozotocin (STZ, 30 mg/kg) intraperitoneally and oral glucose tolerance test was conducted five days later. Animals with >10 mmol/l fasting blood glucose (FBG) or >16.7 mmol/l postprandial blood glucose (PBG) were selected as the diabetic group (DM, n = 48). Animals of chow diet with <7 mmol/l FBG and <10 mmol/l PBG were selected as normal group (NDM, n = 28). Animals were fed on their original diets for another eight weeks after STZ injection.

### Evaluation of Circulating EPCs

Circulating EPCs were quantified on 1, 3, 5, 7, 14 and 28 days after the onset of AMI. Bone marrow-derived early-stage EPCs were defined as CD45^−/low^ mononuclear cells (MNCs) (PE-Cy5.5-conjugated anti-human CD45, Caltag Laboratories) with co-expression of CD34 (FITC-conjugated anti-human CD34, BD Biosciences) and CD133 (PE-conjugated anti-human CD133, Miltenyi Biotech) as well as endothelial specific antigen KDR (APC-conjugated anti-human KDR, R&D Systems). Cells were analyzed by flow cytometry (FACS Canto, Becton Dickinson) and CD45^−/low^/CD34^+^/CD133^+^/KDR^+^ EPCs were expressed as number per 10^6^ MNCs. Rat blood samples were collected on 0 (before surgery), 1, 3, 5, 7, 14 and 28 days after LAD ligation and EPCs were defined as c-kit^+^/CD34^+^ cells per 10^5^ MNCs (PE-conjugated anti-CD34, Santa Cruz, PE-Cy5-conjugated anti-c-kit, eBioscience).

### Determination of Plasma VEGF, SDF-1α and hsCRP Level

Peripheral vein blood sample was collected on 1, 3, 5, 7, 14 and 28 days after the onset of AMI in patients and plasma concentrations of VEGF, SDF-1α and hsCRP were measured by enzyme-linked immunosorbent assay (R&D Systems for VEGF and SDF-1α, Dade Behring Inc. for hsCRP) according to the manufacturer’s instructions.

### Patients Follow Up for Cardiac Function and Cardiovascular Events

Patients were followed up for cardiovascular events and cardiac function. Cardiac function was evaluated by echocardiography afte hospitalization (baseline) and at follow up. The primary endpoints included cardiovascular mortality, re-infarction and nonfatal stroke or transient ischemic attack. Secondary endpoints included: composite cardiovascular primary events including all the three primary endpoints; re-hospitalization for unstable angina and heart failure; composite coronary events including cardiovascular mortality, re-infarction and re-hospitalization; ischemia symptoms occurrence; NYHA scores; coronary stent numbers; coronary revascularization including PCI and CABG; acute coronary thrombosis. All Data were recorded by the collaborating physician on a designed cardiovascular event worksheet.

### Rat Model of Myocardial Infarction

Myocardial Infarction was produced by surgical ligation of the left anterior descending coronary artery (LAD). After anesthetized by ketamine hydrochloride (50 mg/kg) and diazepam (5 mg/kg), chest was opened at the left fourth inter costal space and LAD was ligated by a 6–0 silk suture 1 mm below the tip of the left atrial appendage. Successful ligation was verified by color change and ECG.

### Assessment of Rat Cardiac Function

Four weeks after myocardial infarction, trans-thoracic echocardiography (SONOS 5500, Philips Medical Systems Best, the Netherlands) was used to evaluate rats cardiac function. We measured the left-ventricular end-diastolic diameter (LVIDd), the left-ventricular end systolic diameter (LVIDs), the inter-ventricular septal thickness in diastole (IVST), the LV posterior wall thickness (LVPWT), the systolic ejection fraction (EF) and the percent LV fractional shortening (FS). An observer blinded to the experiment performed the measurements for at least three consecutive pulsation cycles.

### Histological Studies

Hematoxylin and eosin staining was applied to assess cytological structure. Cardiac fibrosis was assessed by Masson’s trichrome staining. The infarct size and fibrosis areas were measured by Image Pro Plus 5.0 software and expressed as a percentage of the total LV. vWF (1∶800, Abcam, USA) and HIF (1∶400, Abcam, USA) immunohistochemistry were applied to assess micro-vessel density and hypoxia degree in the infarct myocardium. We counted 10 random 100× field and mean capillaries per field was obtained for statistical analysis. HIF positive area was measured by Image Pro Plus 5.0 software.

### Western Blot Analysis and Gelatin Zymography

VEGF (mouse-anti VEGF, Santa Cruz), HIF (mouse-anti HIF, Abcam), Akt (rabbit-anti Akt, Cell Signaling), p-Akt (rabbit-anti Akt (ps473), Cell Signaling), eNOS (mouse-anti eNOS/NOS, BD Biosciences), p-eNOS (mouse-anti eNOS (ps1177), BD Biosciences), and MMP-9 (rabbit-anti MMP-9, Abcam) were tested in the bone marrow tissue to assess bone marrow pathway changes after AMI. Expression of proteins and phosphorylation levels was normalized to β-actin (mouse-anti β-actin, Kangchen Biotech) and baseline expression. MMP-9 activity was assessed using the gelatin zymography assay by staining with 0.1% coomassie brilliant blue G-250, 10% acetic acid and 20% methanol and destaining in 10% acetic acid and 20% methanol until bands were visualized. The gel was photographed and quantified by scanning densitometry.

### Statistical Analysis

All categorical variables are expressed as percentages and continuous variables as mean ± SD. We used the chi-square test for comparisons of categorical variables. For continuous variables, comparisons were performed by unpaired Student’s t-test and one-way ANOVA with Dunnett’s post-test. For comparison of cumulative mortality rates, we used Kaplan-Meier survival analysis and the log-rank t test. Multivariate Cox regression with forward selection were used to investigate the relationships between EPCs level, clinical features and cardiovascular mortality. In all cases, a p<0.05 (two-tailed) was considered significance. All analyses were performed using SPSS 17.0 software (SPSS, Inc.).

## Results

### Patient Characteristics

Patients with T2DM had higher FBG and HbA_1C_ levels compared with the non-diabetics (p<0.05). They also smoked less and used more aspirin as well as oral hypoglycemic agents and insulin (p<0.05). GOT was decreased in T2DM patients (p<0.05). No differences were seen in other characteristics between the two groups (Table 1).

**Table 1:** Baseline Characteristics of patients.

	Non-diabetics	Type 2 diabetics	p Value
n	31	31	-
Age(years)	69±9	68±11	0.96
Male	55%	65%	0.44
BMI (kg/m^2^)	24.56±2.79	25.34±2.74	0.27
Hypertension	64%	77%	0.26
Smoking	42%	9%	<0.05^*^
Hyperlipidemia	23%	32%	0.39
CHD family history	9%	13%	0.69
Drugs Before hospitalization			
Statins	0	0	1.00
ACE inhibitors	0	0	1.00
Aspirin	0	23%	<0.05^*^
Beta blockers	23%	13%	1.00
Oral hypoglycemics	0	45%	<0.001^**^
Insulin	0	52%	<0.001^**^
Drugs After hospitalization			
Statins	100%	100%	1.00
ACE inhibitors	100%	100%	1.00
Aspirin	100%	100%	1.00
Beta blockers	100%	100%	1.00
Nitrates	100%	100%	1.00
Oral hypoglycemics	0	45%	<0.001^**^
Insulin	0	52%	<0.001^**^
HbA1c(%)	5.7±0.3	8.0±2.0	<0.05^*^
Glucose (mM)	5.29±0.28	8.51±3.64	<0.05^*^
Cholesterol (mM)	4.31±0.82	4.71±0.76	0.051
Triglycerides (mM)	1.22±0.43	1.33±0.42	0.32
HDL-cholesterol (mM)	0.89±0.33	0.92±0.20	0.67
LDL-cholesterol (mM)	2.36±0.51	2.40±0.71	0.79
Uric acid (mol/l)	355±101	326±88	0.24
Creatinine (mol/l)	83±30	98±29	0.052
GPT (U/L)	33±9	29±10	0.12
GOT (U/L)	185±54	154±38	<0.05^*^

All plasma determinations were performed in the fasting state. BMI: body mass index; CHD: coronary heart disease; ACE: angiotensin-converting enzyme; HDL: high density lipoprotein; LDL: low density lipoprotein; GPT: glutamic-pyruvic transaminase; GOT: glutamic-oxal(o)acetic transaminase.

### Animal Model of Diabetes

FBG, cholesterol and triglyceride levels were higher in rats fed high fat versus chow after 4 weeks (prior to STZ injection) (p<0.05). Eight weeks following STZ injection, FBG and PBG were both higher in the STZ group (p<0.05, Table 2).

**Table 2:** Baseline Characteristics of rats.

	Non-diabeticrats	Type 2 diabeticrats	p Value
n	28	48	–
Weight (g)			
0 week	170.52±9.28	169.39±7.06	0.59
4^th^ week	390.56±47.91	428.21±39.01	0.01^*^
8^th^ week	481.20±14.95	454.55±56.35	0.01^*^
Glucose (mM)			
FBG 4^th^ week	4.6±0.76	6.49±1.01	<0.001^**^
PBG 4^th^ week	5.36±1.16	6.61±1.81	0.15
FBG 8^th^ week	4.52±1.04	12.22±6.69	0.01^*^
PBG 8^th^ week	5.00±1.47	22.38±6.78	<0.001^**^
Cholesterol (mM)	2.03±0.45	2.89±0.69	0.01^*^
Triglycerides (mM)	0.42±0.06	1.57±0.73	<0.001^**^
Insulin (ng/ml)	0.17±0.10	0.15±0.09	0.43

All plasma determinations were performed in the fasting state. FBG: fasting blood glucose PBG: postprandial blood glucose.

### EPCs Mobilization Post-AMI was Suppressed in Patients with T2DM

**Figure 1:**
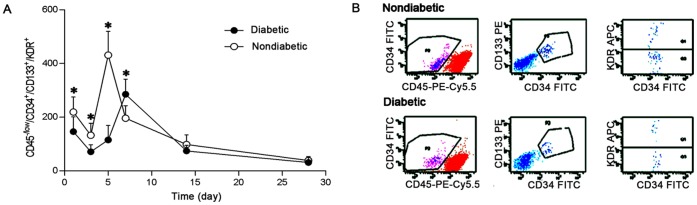
EPCs mobilization following AMI was impaired in diabetics. (A) Mobilization of EPCs in non-diabetics and diabetics at different time points post-AMI (*p<0.05 vs. non-diabetics). (B) Three step analysis by flow cytometry to quantify the CD45^−/low^/CD34^+^/CD133^+^/KDR^+^ EPCs at peak time (non-diabetic: day 5; diabetics: day 7).

In non-diabetic patients, circulating EPCs were found to be high immediately after the onset of AMI (day 1), with a subsequent peak at day 5 and a rapid decline thereafter. A different pattern of EPCs kinetics was observed in patients with T2DM, with similar levels at day 1 but the peak delayed to day 7, and the magnitude of the peak decreased (peak EPCs count 285±56/10^6^ MNCs in diabetics vs. 431±88/10^6^ MNCs in non-diabetics, p<0.05, Figure 1.A). Figures 1.B showed the three-step analysis to quantify circulating CD45^−/low^/CD34^+^/CD133^+^/KDR^+^ EPCs at peak time (non-diabetic day 5; diabetics day 7).

### Plasma VEGF, SDF-1α and hsCRP post-AMI were Higher in Patients with T2DM

**Figure 2:**
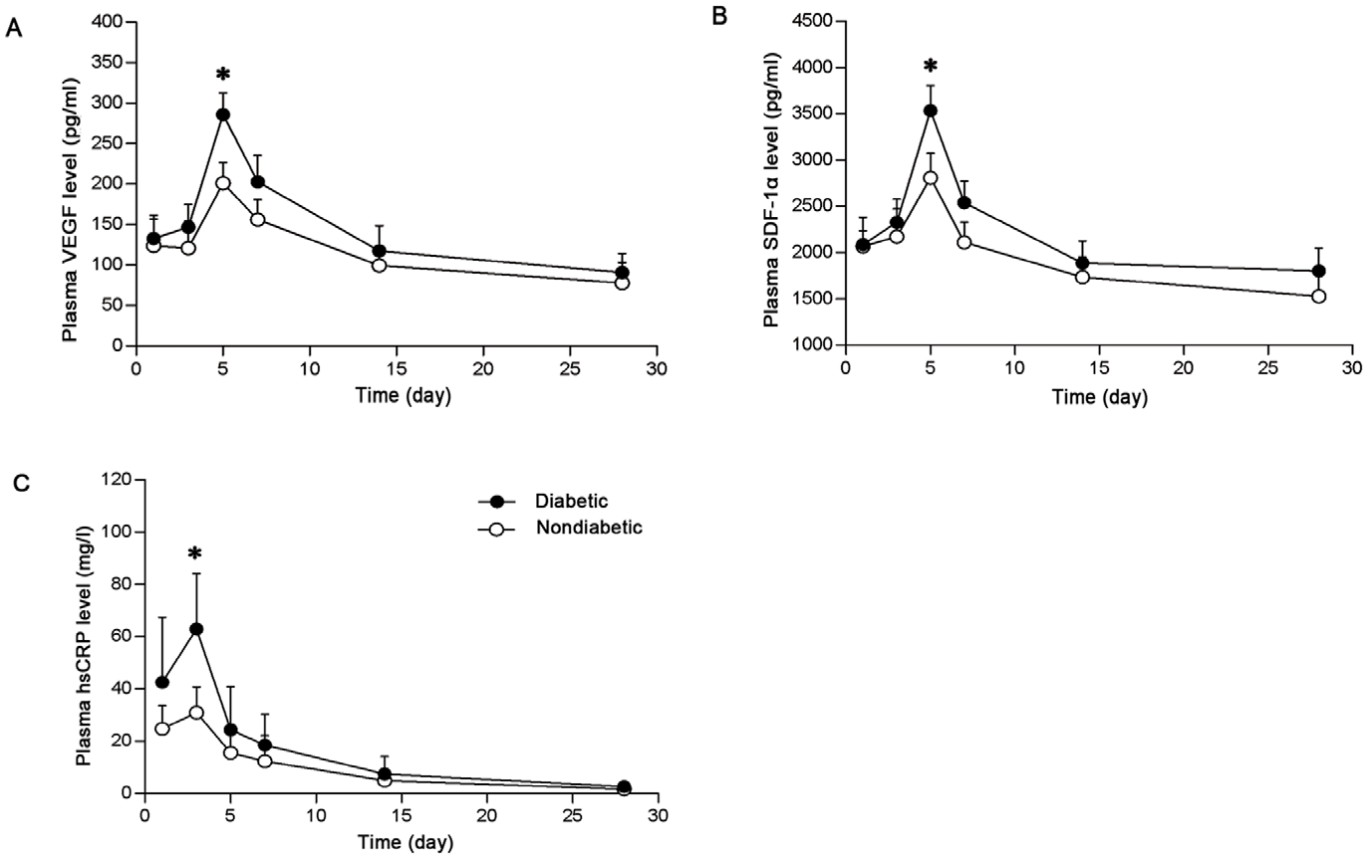
Plasma VEGF, SDF-1α and hsCRP were increased in patients with T2DM post-AMI. Plasma concentrations of (A) VEGF, (B) SDF-1α and (C) hsCRP were assessed by enzyme-linked immunosorbent assay at different time points post-AMI in non-diabetics and diabetics (*p<0.05 vs. non-diabetic patients).

A rapid elevation of plasma VEGF was observed after the onset of AMI in both groups. In non-diabetics, plasma VEGF peaked at day 5 with a gradual decrease thereafter. In T2DM patients, a similar time course was observed but the magnitude of the peak was elevated (Figure 2.A, diabetics vs. non-diabetics, 285±26 pg/ml vs. 201±25 pg/ml, p<0.05). In both groups, plasma SDF-1α reached its highest level on day 5 post-AMI, and the peak level was greater in diabetics (Figure 2.B, diabetics vs. non-diabetics, 3533±269 pg/ml vs. 2807±265 pg/ml, p<0.05). Peak levels of plasma hsCRP were at day 3 post-AMI in both groups, and again the peak was greater in T2DM group (Figure 2.C, diabetics vs. non-diabetics, 63±21 mg/l vs. 31±9 mg/l, p<0.05).

### Patients with T2DM Exhibited Decreased Left Ventricular Function Post-AMI

**Figure 3:**
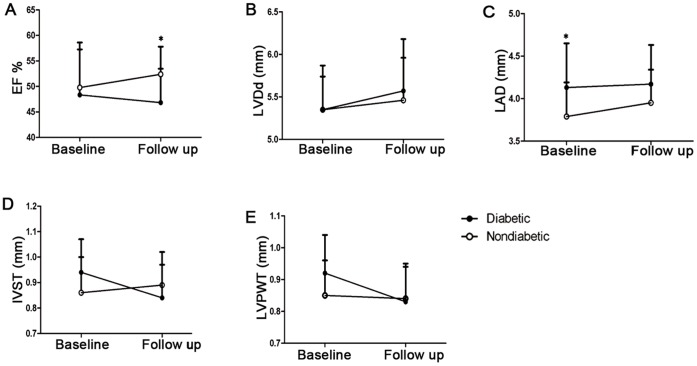
Cardiac function in diabetics and non-diabetics patients with AMI. Baseline echocardiography measurements were taken while hospitalized for AMI. Compared with non-diabetic patients, T2DM patients showed a increased left atrial diameter at baseline and decreased ejection fraction after 2.26 years follow up. (A) Systolic ejection fraction. (*p<0.05 vs. non-diabetic patients at follow up). (B) left ventricular end-diastolic diameter. (C) left atrial diameter. (*p<0.05 vs. non-diabetic patients at baseline). (D) inter-ventricular septal thickness at diastole. (E) left ventricular posterior wall thickness.

The mean time of follow-up was 2.31±1.65 years in diabetics and 2.21±1.82 years in non-diabetics (p>0.05). Cardiac function was evaluated and decreased LVEF was found in T2DM group at follow up (diabetics vs. non-diabetics, 46.83%±6.64% vs. 52.38%±5.42%, p<0.05, Figure 3.A). Other parameters including LVDd, IVST, LVPWT showed no difference, except for an increased LAD in diabetics at baseline (diabetics vs. non-diabetics, 4.13±0.52 mm vs. 3.79±0.40 mm, p<0.05, Figure 3.B–E).

### Clinical Outcome Post-AMI was Worse in Patients with T2DM

**Figure 4:**
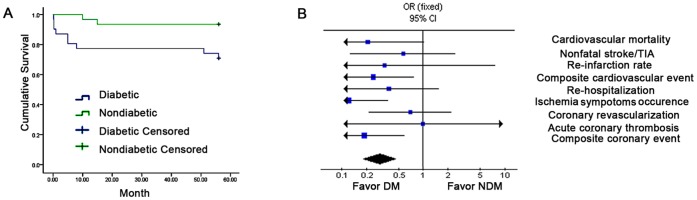
Decreased survival in diabetics during follow up and negative effect of diabetics after AMI. (A) Cumulative survival curve of the two groups at follow up. T2DM patients showed decreased survival rate after AMI. (B) The negative effect of T2DM on clinical outcomes post-AMI.

Clinical outcome at follow up was shown in Table 3. Cardiovascular mortality was greater in patients with T2DM (diabetics vs. non-diabetics, p<0.05), but there was no significant difference in nonfatal stroke/transient ischemic attack or re-infarction rate. Among the secondary end points, composite cardiovascular primary events, recurrence of ischemic symptoms, NYHA score, number of coronary stents and composite coronary events were all higher in T2DM patients (diabetics vs. non-diabetics, p<0.05). However, re-hospitalization, coronary revascularization and acute coronary thrombosis showed no difference between the two groups. The cumulative survival over follow up was lower in diabetics compared with non-diabetics post-AMI (p<0.05, Figure 4.A). The net effect of T2DM on clinical outcomes post-AMI was shown in Figure 4.B. Multivariate Cox regression analysis was performed to determine the independent risk factors for the cardiovascular mortality. Glucose (HR 2.01, 95% CI 1.42–2.85, p = 0.008) emerged as a significant risk factors for the cardiovascular mortality. First day EPC level (HR 0.974, 95% CI 0.952–0.997, p = 0.02) and seven day EPCs level (HR 0.966, 95% CI 0.945–0.988, p = 0.003) were independent prognostic variables.

**Table 3:** Clinical Outcomes after AMI.

Clinical Outcomes	Non-diabetics	Diabetics	Unadjusted Hazard Ratio	p Value
Average follow up time (year)	2.21±1.82	2.31±1.65		0.65
**Primary Endpoints**				
Cardiovascular mortality	6.45%	25.81%	4	<0.05*
Nonfatal stoke/transient ischemic attack	9.68%	16.13%	1.67	0.45
Re-infarction rate	0	3.22%		0.31
**Secondary Endpoints**				
Composite cardiovascular events	16.12%	45.16%	2.80	<0.05*
Re-hospitalization	9.68%	22.58%	2.33	0.16
Ischemia symptoms occurrence	32.25%	80.65%	2.50	<0.05*
NYHA score	1.75±0.68	2.21±0.63	1.26	<0.05*
Coronary stent number	1.53±0.64	2.23±0.82	1.46	<0.05*
Coronary revascularization procedures	77.42%	80.65%	1.04	0.76
Acute coronary thrombosis	3.22%	3.22%	1	1.00
Composite coronary events	16.13%	51.61%	3.2	<0.05*

Unadjusted hazard ratio is calculated as the incidence in diabetics divided by the incidence in non-diabetics.

### Decreased EPCs Mobilization and Impaired Bone Marrow HIF/VEGF/Akt/eNOS/MMP-9 Signaling in Diabetic Rats Post-AMI

**Figure 5:**
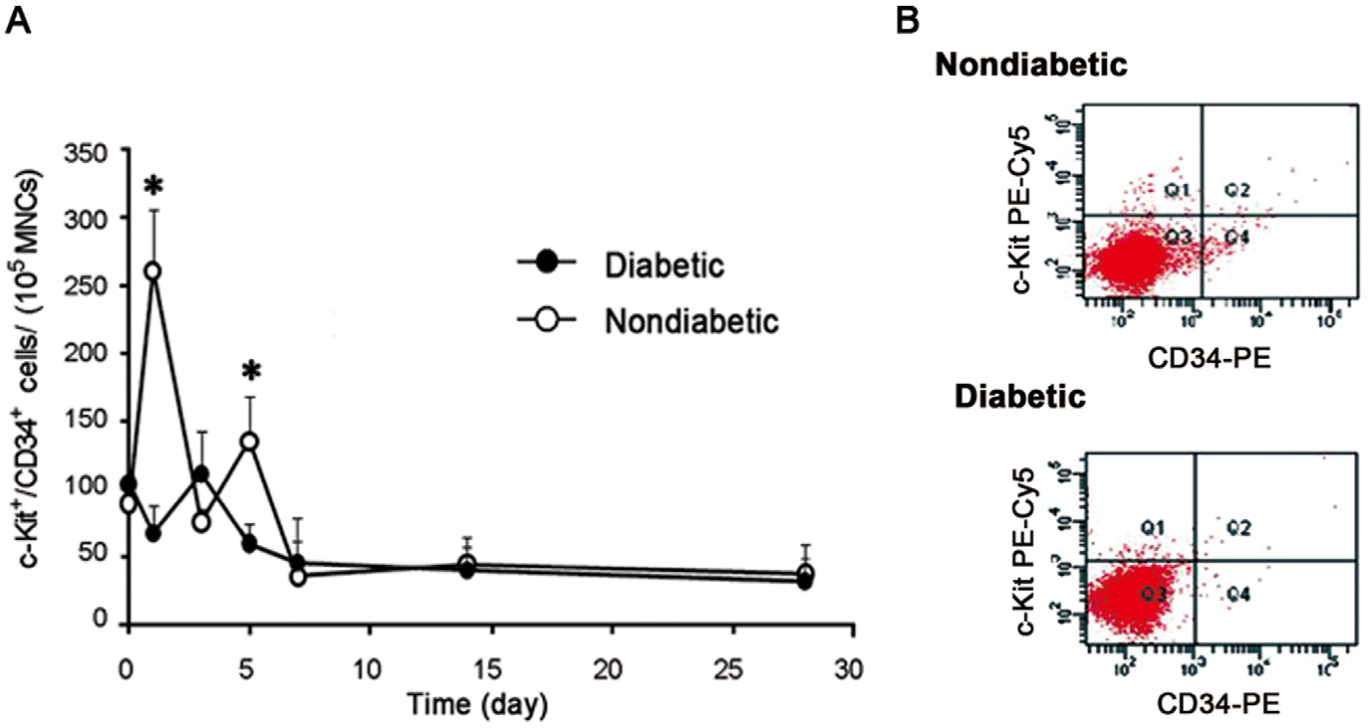
EPCs mobilization in rats post-AMI. (A) Mobilization of EPCs after myocardial infarction in rats at different time time points post-AMI (*p<0.05 vs. non-diabetic rats). (B) Flow cytometry analysis of the CD34^+^/c-Kit^+^ EPCs at peak time.

**Figure 6:**
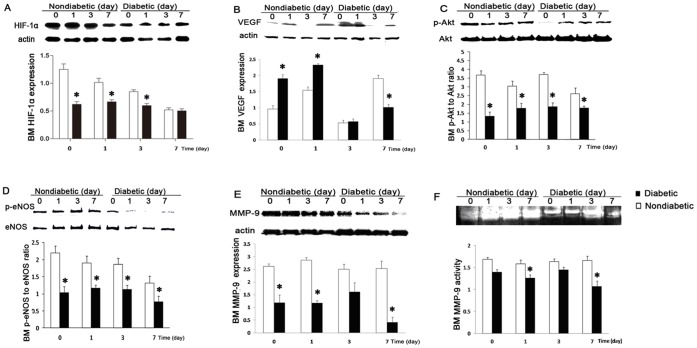
Bone marrow signalling impairment in diabetic rats post-AMI. Proteins expression in bone marrow at days 0, 1, 3, 7 after myocardial infarction were measured by western blot. (A) HIF (B) VEGF (C) phospho-Akt to Akt (D) phospho-eNOS to eNOS (E) MMP-9 (F) MMP-9 activity measured by gelatin zymography (*p<0.05 vs. non-diabetic rats).

Non-diabetic rats exhibited two peaks of circulating EPCs, the first at day 1 and the second at day 5, whilst the diabetic rats exhibited no clear peak and a lower level of EPCs on both days 1 and 5 (Figure 5.A-5.B). Western blot showed that bone marrow VEGF protein expression was higher in diabetic rats on days 0 and 1 post-AMI but dropped greatly on day 3 and 7, while phosphorylation of Akt and eNOS as well as HIF were decreased in diabetic rats at all time points (p<0.05, Figure 6.A-6.D). Diabetes was also associated with lower bone marrow MMP-9 expression on day 0, 1 and 7 after AMI and decreased MMP-9 activity on day 1 and 7 (p<0.05, Figure 6.E-6.F).

### Cardiac Function Post-AMI is Worse in Diabetic Rats

**Figure 7:**
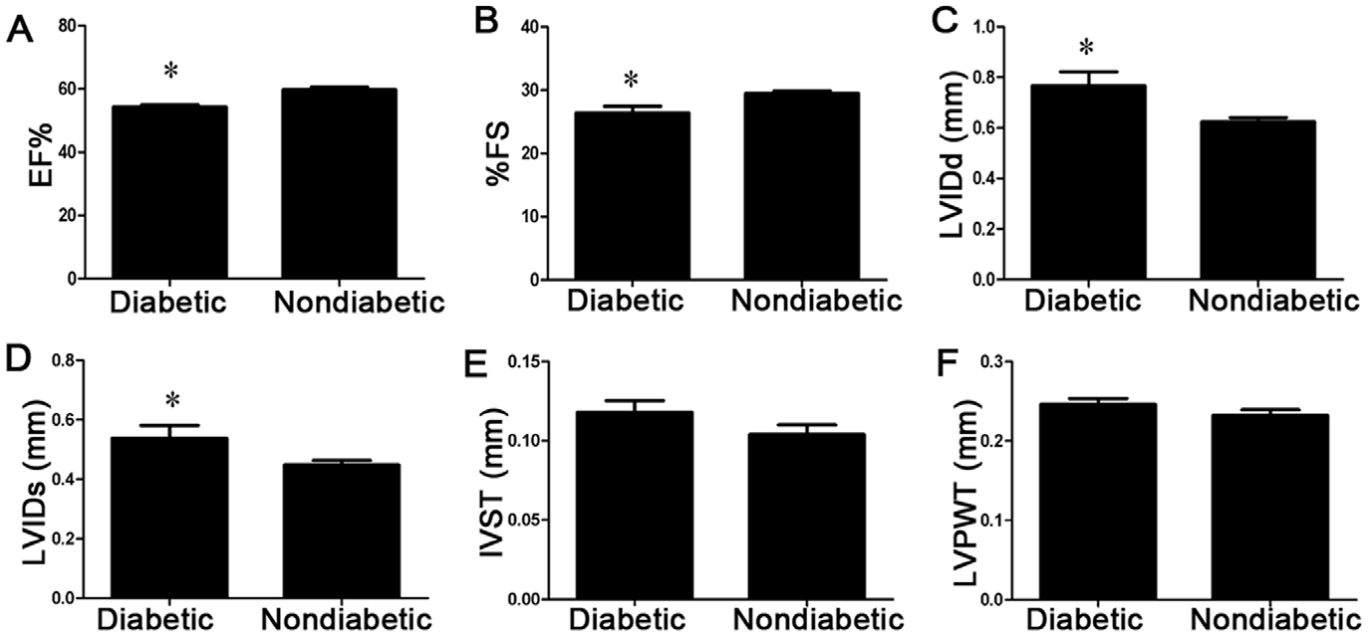
Echocardiography showed deceased cardiac function in diabetic rats four weeks after AMI. (A) Systolic function index ejection fraction (*p<0.05 vs. non-diabetic rats). (B) Systolic function index percent fractional shortening (*p<0.05 vs. non-diabetic rats). (C) Left ventricle end-diastolic diameter (*p<0.05 vs. non-diabetic rats). (D) Left ventricle end-systolic diameter (*p<0.05 vs. non-diabetic rats). (E) The inter-ventricular septal thickness at diastole. (F) Left ventricle posterior wall thickness.

Four weeks after AMI, diabetic rats exhibited decreased EF and %FS (diabetics vs. non-diabetics, EF 54.25%±1.58% vs. 59.72%±1.89%; %FS 26.41%±2.31% vs. 29.47%±0.89%, Figure 7.A-7.B, p<0.05). LVIDd and LVIDs were both higher in the diabetic group, suggesting left ventricular enlargement (diabetics vs. non-diabetics, LVIDd (mm) 0.76±0.12 vs. 0.62±0.03; LVIDs (mm) 0.54±0.08 vs. 0.44±0.04, Figure 7.C-7.D, p<0.05). IVST and LVPWT showed no difference between the two groups (Figure 7.E-7.F).

### Diabetic Rats Exhibited Increased Left Ventricular Remodeling and Decreased Angiogenesis

**Figure 8:**
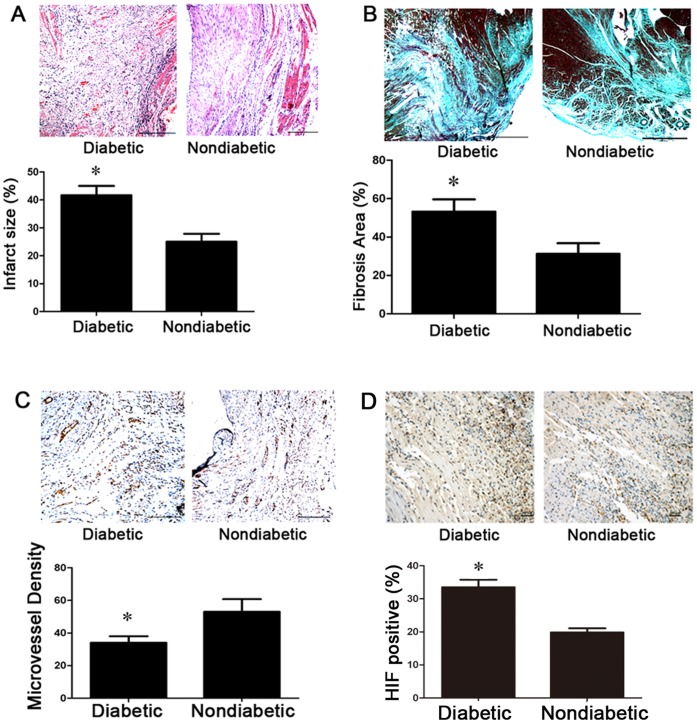
Cardiac morphology showed increased remodelling in diabetic rats. (A) Infarct myocardium stained with hematoxylin-eosin (*p<0.05 vs. non-diabetic rats). (B) Masson’s trichrome staining showed the fibrosis area (*p<0.05 vs. non-diabetic rats). (C) vWF immuno-histochemistry of micro-vessels (*p<0.05 vs. non-diabetic rats). (D) HIF immuno-histochemistry staining of the myocardium (*p<0.05 vs. non-diabetic rats).

Hematoxylin-eosin staining showed enlarged infarct size in diabetic rats post-AMI (diabetics vs. non-diabetics, 41.67%±3.33% vs. 25.00%±2.88%, p<0.05, Figure 8.A). Hearts from diabetic rats exhibited increased area of interstitial fibrosis (diabetics vs. non-diabetics, 53.25%±6.36% vs. 31.25%±5.54%, p<0.05, Figure 8.B) and decreased microvessel density compared with non-diabetic rats (diabetics vs. non-diabetics, 34±4.11 vs. 53±7.75, p<0.05, Figure 8.C). Immuno-histochemistry staining showed increased expression of HIF in diabetic rats post-AMI (diabetics vs. non-diabetics, 33.50%±2.27% vs. 19.83%±1.25%, p<0.05, Figure 8.D).

## Discussion

Clinical outcome post-AMI was worse in patients with T2DM than in non-diabetic patients [27]. The mechenism underlying this remains unclear. Mobilization of EPCs from bone marrow after AMI play an key role in myocardium repair by facilitating new vessel formation and endothelium function maintenance [8], [9].

In the present study we observed the kinetics of circulating EPCs level after AMI in T2DM patients. Compared with non-diabetic AMI patients, circulating EPCs level was delayed in peak time and decreased in peak level in T2DM AMI patients, with a higher plasma level of VEGF, SDF-1α and hsCRP. We followed up these patients and found that at an average of 2.26 years, T2DM patients had higher cardiovascular composite events compared with non-diabetic patients and showed deteriorated left ventricle function. Multivariate Cox regression analysis showed that among the various clinical features, blood glucose was a significant risk factor for cardiovascular mortality, while EPCs level on day 1 and day 7 emerged as independent prognostic variables. This was in accordance with previous researches by Hill et al, who proved that levels of endothelial progenitor cells may be a surrogate biologic marker for vascular function and cumulative cardiovascular risk [13]. The same change of EPCs mobilization was observed in an AMI and T2DM rat model, with deteriorated cardiac function, larger infarct size and fibrosis degree, more severe hypoxia degree as well as decreased micro-vessels density. However, in the rat model, the link between impaired EPCs mobilization and progressed left ventricle deterioration was not a direct one and we may extend from the clinical part of our study that insufficient EPCs mobilization in rat may partly clarify the progressed left ventricle remodeling and worsened cardiac display. We also explained the underlying mechanism by assessing the change in bone marrow HIF/VEGF/Akt/eNOS/MMP-9 pathways and observed an lower phosporylation level of Akt, eNOS, HIF and decreased expression and activity of MMP-9 in T2DM rats immediately after AMI. Bone marrow VEGF was higher in T2DM rat acutely after AMI and dropped quickly.

Elevated VEGF has been shown to mobilize bone marrow EPCs into the peripheral circulation [4]. After AMI, local ischemia in the infracted myocardium induces up regulation of VEGF via the hypoxia-inducible factor (HIF) activation, and subsequently stimulates nitric oxide (NO) synthesis and downstream MMP-9 activation in the bone marrow [28], [29]. The activation of MMP-9 in turn induces EPCs mobilization by facilitating transformation of membrane-bound kit ligand to soluble kit ligand [30], [31]. Besides VEGF, SDF-1α is also vital in EPCs mobilization and homing. Elevated SDF-1α is able to induce the chemotaxis of EPCs via the HIF pathway by binding to the CXCR4 [32], [33]. Defective mobilization of EPCs in diabetic rats is associated with insufficient release of SDF-1α [34], [35]. Here we found that the suppressed circulating EPCs level was accompanied by augmented up regulation of plasma VEGF and SDF-1α after AMI in T2DM patients. This elevation may be a reaction to the severer ischemia situation in T2DM and may act to drive more EPCs into the peripheral blood. However, despite the high level of cytokines in T2DM patients, circulating EPCs was not elevated subsequently, suggesting an obstruction in EPCs mobilization signaling.

Many signaling pathways are involved in the regulation of EPCs mobilization and homing. The PI3K/Akt/eNOS pathway has been implicated in inducing the mobilization of EPCs from the bone marrow, promoting EPCs differentiation and inhibiting EPCs apoptosis [24], [36]. NO is also an essential requirement in EPCs migration, homing and formation of neovascularization. T2DM patients are always accompanied by insulin-resistant, which reduced the bioavailability of NO and consequently hindered the activation of MMP-9 [32]. In this study, we observed an impaired HIF/Akt/eNOS/MMP-9 pathway in the bone marrow of AMI rat with T2DM. Bone marrow phosporylation level of Akt, eNOS and HIF were inhibited and the expression and activity of MMP-9 was both decreased, which may directly influence the survival of EPCs and hinder EPCs mobilization from bone marrow into peripheral circulation. This may be the underlying mechanism for the poor mobilization of EPCs in the presence of high plasma VEGF and SDF-1 in T2DM patients.

CRP has emerged as an important inflammatory marker with a strong predictive value for cardiac events [37]. We found that there was an up regulation of plasma hsCRP in T2DM patients after AMI. This was reasonable as the severer ischemia or hypoxia condition in T2DM may lead to a stronger inflammation reaction. Meanwhile, the insulin-resistant condition in T2DM was also a low-level inflammation, which resulted in increased levels of CRP in response and may subsequently change EPCs quantity and function. It was found that CRP at concentrations significantly reduced EPCs cell number and increased EPCs apoptosis [38]. Conversely, a transient inflammatory condition was proved to stimulate expression of adhesion molecules and chemotactic factors through activation of NF-κB in the endothelium and facilitate EPCs release and homing [39]. Further investigations are needed to address whether the net effect of CRP in inflammatory condition is to facilitate or to hinder EPCs mobilization.

This study has limitations. C-kit^+^ cells were believed to contain cellular subpopulations that had the potential to give rise to EPCs and CD34 was expressed on the surface of lymphohematopoietic stem and progenitor cells. In our study, we assessed the number of c-kit^+^/CD34^+^ cells in rats, which may represent mostly hematopoietic progenitor cells from the bone marrow. Although this group of cells were able to give rise to EPCs and presented bone marrow progenitor cells mobilization after acute myocardial infarction in rats, extra endothelial lineage antigen like Flk1 or CD31 were recommened in further study.
